# Effects of Activin and TGFβ on p21 in Colon Cancer

**DOI:** 10.1371/journal.pone.0039381

**Published:** 2012-06-26

**Authors:** Jessica Bauer, Judith C. Sporn, Jennifer Cabral, Jessica Gomez, Barbara Jung

**Affiliations:** 1 Department of Medicine, Northwestern University Feinberg School of Medicine, Chicago, Illinois, United States of America; 2 Department of Medicine, University of California San Diego, La Jolla, California, United States of America; Rush University Medical Center, United States of America

## Abstract

Activin and TGFβ share SMAD signaling and colon cancers can inactivate either pathway alone or simultaneously. The differential effects of activin and TGFβ signaling in colon cancer have not been previously dissected. A key downstream target of TGFβ signaling is the cdk2 inhibitor p21 (p21^cip1/waf1^). Here, we evaluate activin-specific effects on p21 regulation and resulting functions. We find that TGFβ is a more potent inducer of growth suppression, while activin is a more potent inducer of apoptosis. Further, growth suppression and apoptosis by both ligands are dependent on SMAD4. However, activin downregulates p21 protein in a SMAD4-independent fashion in conjunction with increased ubiquitination and proteasomal degradation to enhance migration, while TGFβ upregulates p21 in a SMAD4-dependent fashion to affect growth arrest. Activin-induced growth suppression and cell death are dependent on p21, while activin-induced migration is counteracted by p21. Further, primary colon cancers show differential p21 expression consistent with their *ACVR2/TGFBR2* receptor status. In summary, we report p21 as a differentially affected activin/TGFβ target and mediator of ligand-specific functions in colon cancer, which may be exploited for future risk stratification and therapeutic intervention.

## Introduction

Activin is a member of the TGFβ superfamily that regulates cell differentiation, proliferation, and apoptosis in many epithelial and mesenchymal cells [1]. Similar to TGFβ, activin utilizes two types of surface receptors with intracellular SMAD2, 3 and 4 for signal transduction. Activin receptor 1 (ACVR1B) and activin receptor 2 (ACVR2) are transmembrane proteins with extracellular ligand-binding activity and intracellular serine/threonine kinase activity. ACVR2B does not substitute for the functions and signaling of ACVR2 [2].

In particular, *ACVR2* was found mutated in the majority of colorectal cancers with high frequency microsatellite instability (MSI-H), primarily due to a frameshift in the A_8_ tract of exon 10 [3], [4]. Restoration of activin signaling, its growth suppression, growth arrest and its induction of migration occur when *ACVR2* is complemented [5]. We have previously demonstrated high frequency of *ACVR2* mutations in MSI-H colon cancer specimens in conjunction with loss of ACVR2 protein expression [6] and showed that ACVR2 loss is associated with larger colon tumors and poor histologic grade [7]. Both *ACVR2* and *TGFBR2* mutations commonly occur simultaneously in MSI cancers [6], and cell lines also can lose both TGFβ and activin signaling [8]. Interestingly, both receptors are less commonly inactivated in MSS colon cancers, which tend to have a worse prognosis than MSI-H colon cancers [9], and both pathways may be targeted independently. To date, little is known about the distinct contribution of activin signaling to colon cancer development and metastasis and specifically, how TGFβ and activin signaling effects differ despite identical intracellular SMAD signaling.

p21 (also known as p21^cip1/waf1^) is a cyclin-dependent kinase inhibitor controlling cell cycle arrest via cdk1 and 2 inhibition and is a master regulator of multiple tumor suppressor pathways via both p53-dependent and independent mechanisms [10]. It is a known target gene of TGFβ in colon cancer [11], and has been associated with activin-induced growth arrest in plasmacytic and breast cancer cells [12], [13], but effects of activin on p21 in colon cancer cells as well as downstream consequences have not been assessed.

In this study, we explored the mechanisms of TGFβ and activin on p21 regulation and the ensuing functional effects thereof in colon cancers. We found that despite identical intracellular SMAD signaling, TGFβ and activin regulate p21 via diverse mechanisms that are functionally relevant in colon cancer leading to more apoptosis or reduction in growth suppression dependent on the activin/TGFβ signaling status with p21 as a differentially regulated target.

## Results

### In the Presence of SMAD4, TGFβ is a more Potent Inducer of Growth Suppression While Activin is a more Potent Inducer of Apoptosis

**Figure 1:**
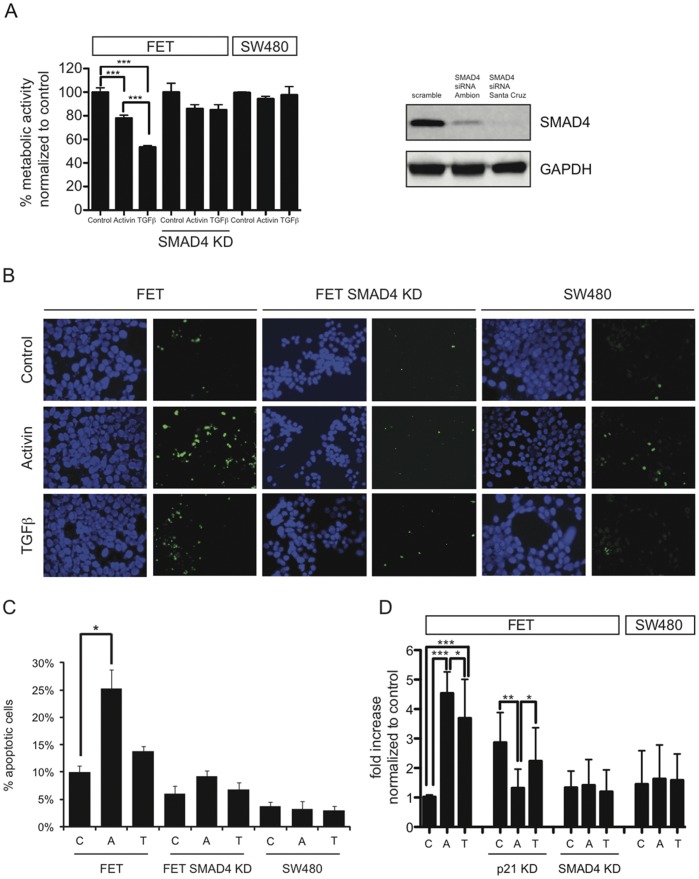
In the presence of SMAD4, TGFβ is a more potent inducer of growth suppression and activin a more potent inducer of apoptosis. A) After *ACVR2/TGFBR2/SMAD4*-wild type FET, FET cells following transient SMAD4 knockdown, and *ACVR2/TGFBR2*-wild type/*SMAD4*-null SW480 cells were treated with vehicle (control), activin or TGFβ for 24 hours, the metabolic activity via MTT-growth assay was assessed. Growth suppression occurred only in the presence of SMAD4 following both activin and TGFβ treatment (***p<0.001). Further, TGFβ led to a significant increase in growth suppression compared to activin (***p<0.001). B) To determine the rate of apoptosis, a TUNEL assay was performed in *SMAD4*-wild type FET, *SMAD4*-KD FET, and *SMAD4*-null SW480 cells treated with control vehicle (C), activin (A), or TGFβ(T). Apoptosis was determined by TUNEL-labeling of apoptotic bodies. C) Normalization [% apoptotic bodies/nuclei] revealed that activin- and TGFβ-induced apoptosis occurred predominantly in *SMAD4*-wild type FET and not in *SMAD4*-KD FET or *SMAD4*-null SW480 colon cancer cells. In the SMAD4 expressing cells, activin induced apoptosis to a greater degree than TGFβ (*p<0.05). D) BrdU-labeled intracellular DNA fragments, indicative of apoptosis, were determined 24 hour after activin or TGFβ treatment of *SMAD4*-wild type FET colon cancer cells, FET cells with p21 KD, FET cells with SMAD4 KD and *SMAD4*-null SW480 colon cancer cells. Increase in DNA fragmentation was noted after activin and TGFβ treatment only in the SMAD4 wild type cells, with activin inducing more fragmentation compared to TGFβ. In the presence of SMAD4, p21KD lead to a basal increase in apoptosis, but activin treatment lead to no induction of apoptosis. SMAD4 knockdown resulted in loss of apoptosis in FET cells akin to effects observed in the *SMAD4*-null SW480 cells (*p<0.05, **p<0.01, ***p<0.001).

To test and compare the effects of activin and TGFβ on cell growth, we used colon cancer cell lines with differing SMAD4 status as described elsewhere [22], [23] in addition to SMAD4 knockdown. The *ACVR2*/*TGFBR2/SMAD4* wild type microsatellite stable colon cancer cell line FET and *ACVR2/TGFBR2* wild type/*SMAD4*-null SW480 colon cancer cells were treated with either activin or TGFβ and cellular growth was assessed. As an additional control, SMAD4 was knocked down via siRNA in FET cells which were treated and analyzed accordingly. While both activin and TGFβ treatment led to significant growth suppression in *SMAD4* wild type FETs, the effect was more pronounced following TGFβ treatment. In contrast, neither ligand was growth suppressive in the absence of SMAD4 in *ACVR2/TGFBR2* wild type/*SMAD4*-null SW480 colon cancer cells or following SMAD4 knockdown in *SMAD4* wild type FET cells (**Figure 1A**).

We then compared apoptosis induction of either ligand in the presence and absence of SMAD4 or p21. Activin induced apoptosis to a greater extent than TGFβ, and apoptosis only occurred in the presence of SMAD4 (**Figure 1B, C**). SMAD4/p21 dependence was confirmed by an alternative apoptosis assay determining BrdU-labeling of intracellular DNA fragments. Apoptosis was increased following activin and TGFβ treatment in SMAD4 positive FET cells, with activin inducing a greater degree of apoptosis. No induction of apoptosis with either ligand was observed in *SMAD4*-null SW480 cells or FET cells following SMAD4 knockdown paralleling the TUNEL experiments (**Figure 1B, C**). p21 knockdown in *SMAD4* wild type FET cells resulted in loss of apoptosis induction (**Figure 1D**).

In conclusion, this data suggests that although activin and TGFβ share intracellular SMAD signaling, each favors distinct downstream physiologic effects at consistent doses. Additionally, we show that both growth suppression and apoptosis induced by either ligand are SMAD4-dependent.

### Activin Regulates Nuclear p21 in a SMAD4-independent Manner

**Figure 2:**
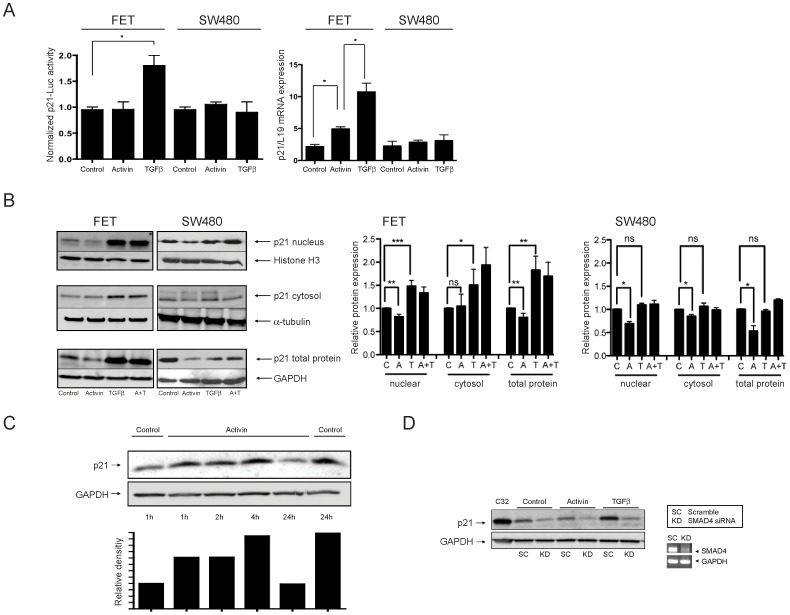
While TGFβ increases p21 expression in the presence of SMAD4, activin decreases nuclear and total p21 independent of SMAD4 status. A) *SMAD4*-wild type FET and *SMAD4*-null SW480 colon cancer cells were treated with vehicle (control), activin, or TGFβ for 24 hours. p21-specific transactivation was determined using a dual luciferase assay with pWWP-luc and pRL-TK (left panel) and mRNA expression levels of p21 were quantified by qPCR and normalized to L19 (right panel). While TGFβ markedly induced both p21-specific transactivation and transcription in the presence of SMAD4, no increase in p21 transactivation and only a modest increase in transcription following activin treatment in the presence of SMAD4 were found (*p<0.05). B) *SMAD4*-wild type FET and *SMAD4*-null SW480 cells were treated with control vehicle (C), activin (A), TGFβ(T), or a combination of both ligands (A+T) for 24 hours prior to lysis for total protein, nuclear, and cytoplasmic preparation. Histone H3, α-tubulin, and GAPDH were used as loading controls for the respective fractions. While TGFβ markedly increased p21 levels in all three fractions in the SMAD4 positive cell line only, activin induced a decrease in nuclear and total p21 protein in SMAD4-positive and -negative cells (left panel). Densitometric analysis of all blots revealed statistically significant changes in p21 levels (right panel) (ns  =  non-significant, *p<0.05, **p<0.01, ***p<0.001). C) Initial upregulation of p21 protein is followed by downregulation by 24 h after activin treatment. *SMAD4*-wild type FET cells were treated with activin or vehicle (control) and harvested at various time points for quantification of p21 protein expression. GAPDH was used as loading control and relative expression was calculated via densitometry. D) While TGFβ-induced upregulation of p21 was SMAD4 dependent, activin-induced downregulation of p21 was still observed in the absence of SMAD4. *SMAD4*-wild type FET cells were treated with vehicle (CNT), activin or TGFβ in the presence of either scramble siRNA (SC) or SMAD4 siRNA (KD) and total p21 levels were determined. GAPDH was used as loading and C32 cell lysate as p21 positive control.

One of the known growth suppressive target genes of TGFβ is p21, which is upregulated following TGFβ treatment in FET colon cancer cells [11]. The effect of activin on p21 in colon cancer has not been assessed. To analyze the downstream effects of SMAD4-dependent activin signaling, we determined p21 expression following activin treatment compared to TGFβ treatment. Contrary to the previously known TGFβ effects on p21, we found no increase in p21 transactivation and only a modest increase in transcription following activin treatment in the presence of SMAD4, while TGFβ markedly induced both p21-specific transactivation and transcription when SMAD4 was present (**Figure 2A**). With regard to p21 protein expression, we found that in contrast to TGFβ, activin treatment decreased nuclear and total p21 regardless of the presence of SMAD4, while cytosolic p21 remained relatively constant (**Figure 2B**). To further analyze the regulation of p21 protein by activin, we performed a time course showing that after slight initial upregulation, p21 protein is downregulated by 24 hours following activin treatment (**Figure 2C** **, two adjacent right lanes**).

To confirm that the ligand effects on p21 were directly dependent on SMAD4, we knocked down SMAD4 in *SMAD4* wild type FET colon cancers cells using siRNA. We found that baseline p21 expression in FET cells decreased with SMAD4 knockdown (**Figure 2D**, lane 3), which substantiates the importance of the SMAD4 pathway for the maintenance of high p21 levels in this cell line [11]. Consistently, TGFβ-induced upregulation of p21 was abolished with loss of SMAD4 (**Figure 2D** **,** lane 7). As expected, the downregulation of p21 by activin was not affected by the absence of SMAD4 (**Figure 2D**, lane 5) which is consistent with our Western blot analysis of p21 levels in FET and SW480 cells (**Figure 2B**) showing downregulation of p21 in the SMAD4 positive and negative cell line. Thus, SMAD4 signaling appears to be required for processes that are dependent on high p21 expression, but dispensable for processes associated with low or decreased p21 levels.

### Activin-induced Growth Suppression is Dependent on p21 Expression, and SMAD4/p21 Signaling can Counteract Activin-induced SMAD4-independent Migration

**Figure 3:**
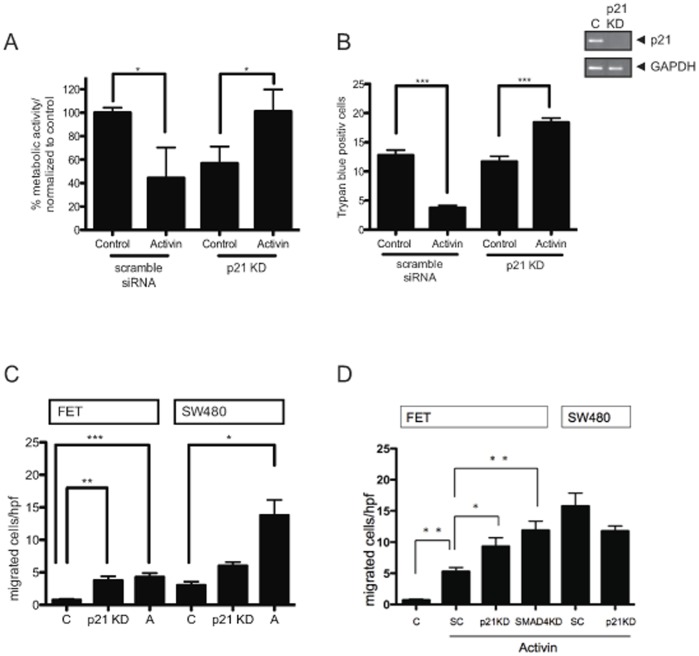
p21 mediates activin-induced growth suppression and counteracts activin-induced SMAD4-independent migration in the presence of SMAD4. A) FET cells were treated with either scramble (SC) or p21 specific siRNA (KD). Growth suppression was assessed by MTT-metabolic assay following activin treatment. Activin induced cell growth inhibition in the presence of p21, but the effect was reversed in the absence of p21 (*p<0.05). B) Total viability is decreased in SMAD4 wild type colon cancers following activin treatment in the presence of p21. FET cells were treated with either scramble or p21 specific siRNA. Cell viability was assessed by trypan blue staining following activin treatment. Trypan blue positive cells after activin treatment were decreased in presence of p21, but increased after p21 knockdown (***p<0.001). C) Activin (A) induces cell migration in SMAD4-positive and SMAD4-negative cell lines. Cellular migration is induced in *SMAD*4-wild type FET cells and *SMAD4*-null SW480 cells following activin treatment, but more pronounced induction of migration is seen in the absence of SMAD4. Loss of p21 leads to an increase in baseline migration in SMAD4 expressing cells (*p<0.05, **p<0.01, ***p<0.001). D) p21 knockdown increases the overall pro-migratory effect of activin in FET cells. Loss of p21 in the absence of SMAD4 does further increase migratory induction (*p<0.05, ** p<0.01).

Subsequently, we sought to determine the functional role of p21 in activin-induced growth suppression and cell viability. We found that loss of p21 via siRNA knockdown resulted in abolishment of activin-induced growth suppression in *SMAD4-*wild type cells, indicating that activin/SMAD4-induced growth suppression is p21-dependent (**Figure 3A**). Further, loss of p21 not only led to abrogation of activin-induced cell death, but also to an increase in cell numbers suggesting a survival benefit with loss of p21 (**Figure 3B**). These observations underscore the importance of the SMAD4/p21 axis in activin-mediated growth suppression and cell death.

Accordingly, we tested the role of the p21 in activin-induced migration by assessing cellular mobility in the presence and absence of SMAD4. We found that activin enhanced migration in SMAD4 positive and SMAD4 negative cells (**Figure 3C**), which argues for a SMAD4 independent pathway regulating migration. This data is supported by similar findings in TGFβ signaling for which a strong pro-migratory and SMAD4-independent effect was shown [20], [22]. As activin treatment was associated with a decrease in p21 levels as well an increase in migration, we expected that loss of p21 by knockdown would enhance baseline migration as well as migration after activin treatment in SMAD4 intact cells, if the remaining p21 was at least partially involved in counteracting activin-induced migration. Consistent with this hypothesis, we show an increased basal migration rate in SMAD4 expressing cells following p21 knockdown (**Figure 3C**) as well as overall more pronounced migration upon activin treatment (**Figure 3D**).

**Figure 4:**
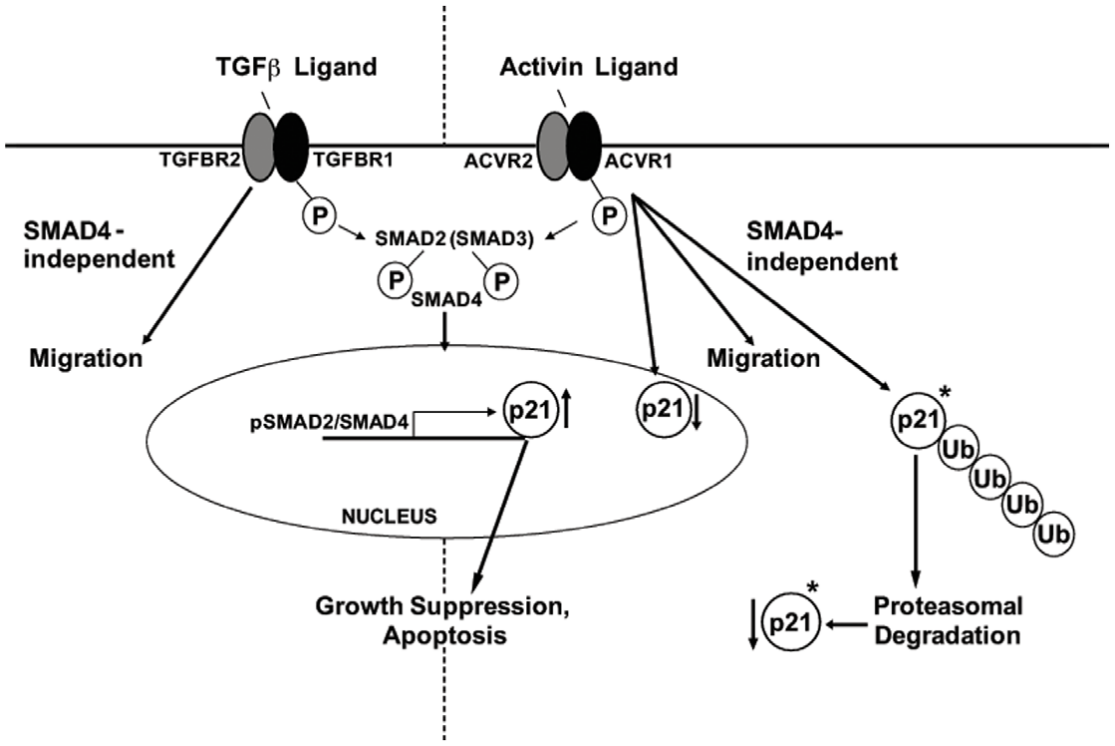
Schematic of proposed differential regulation and effects of activin and TGFβ signaling on p21 in colon cancer cells. * is indicative of total (cytoplasmatic + nuclear) p21.

We further found that basal cell migration was enhanced by activin treatment in the absence of either p21 or SMAD4 in SMAD4-positive FET cells, but that knockdown of p21 had no additional effect on migration when SMAD4 was absent, as in SW480 cells (**Figure 3D** **)**. For TGFβ, we found that cell migration was enhanced regardless of the presence of p21 (**Figure 2B** **,** **Figure S1D**). This supports again that p21-mediated effects following activin treatment are dependent on SMAD4 and that p21 acts downstream of SMAD4 for its anti-proliferative and anti-migratory effects (**Figure 4**). Further, this suggests that, in the case of TGFβ, some promigratory signals can bypass SMAD4 as previously postulated [22] circumventing p21 and its inhibitory effects. In summary, we deduce that p21 may counteract migration downstream of SMAD4 and that downregulation of p21 may be responsible for some of the pro-migratory potential of activin signaling. Thus, differential regulation of p21 may be at the center of distinct functional effects of activin and TGFβ.

### Activin Treatment Leads to Ubiquitination of p21 and Inhibition of Proteasome Abolishes Activin-induced p21 Downregulation

**Figure 5:**
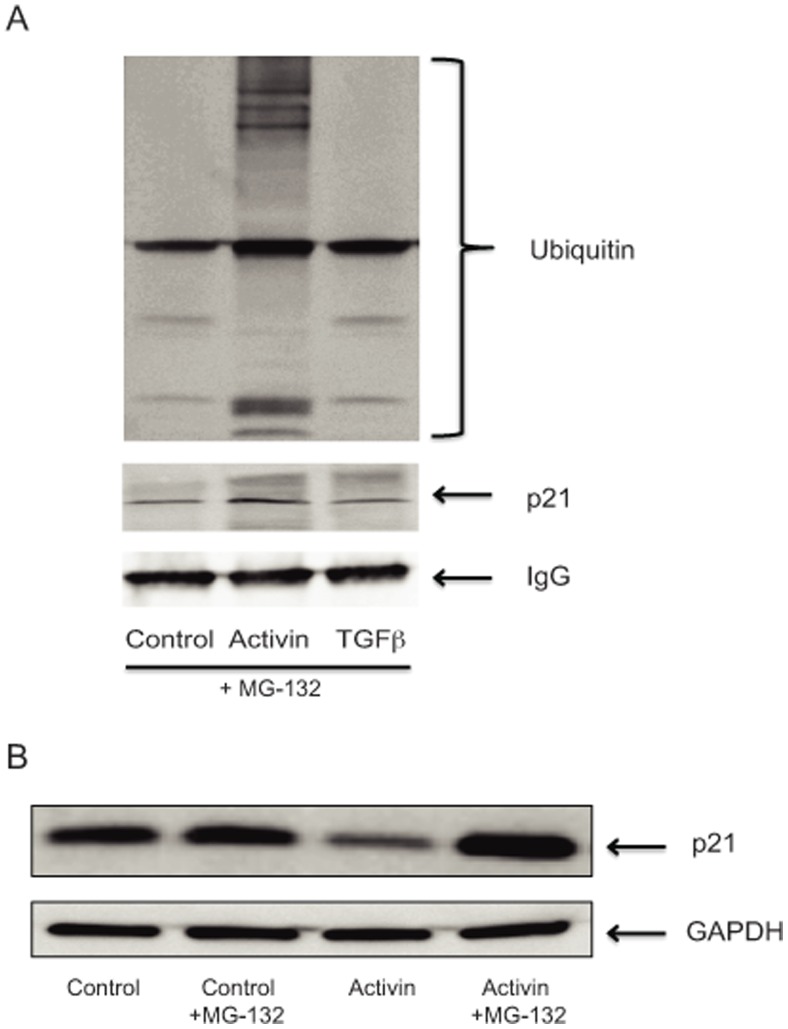
Activin-induced p21 downregulation is associated with ubiquitination and counteracted by proteasomal degradation. A) *ACVR2/TGFBR2/SMAD4*-wild type FET cells were were pretreated for 30 minutes with proteasomal inhibitor MG-132 and then treated with vehicle (control), activin, TGFβ for 24 hours and ubiquitination of total p21 was assessed via immunoprecipitation of p21 and blotting with a ubiquitin-specific antibody (upper panel) and reblotting of p21. Multiple bands indicative of polyubiquitination were seen only following activin treatment. B) Activin-induced p21 downregulation is dependent on the proteasome. *SMAD4*-wild type FET cells were pretreated for 30 minutes with proteasomal inhibitor MG-132 followed by treatment with vehicle (control) or activin for 24 hours and compared to cells treated accordingly without proteasomal inhibition. p21 expression was assessed and showed inhibition of p21 downregulation following activin treatment in conjunction with proteasomal inhibition.

To further dissect the mechanism of activin-mediated p21 protein decrease, we assessed p21 ubiquitination following activin treatment and its dependence on the proteasome (**Figure 5A, B**). For this, we compared p21 ubiquitination following activin and TGFβ treatment. In contrast to TGFβ, activin treatment induced p21 polyubiquitination (**Figure 5A**). Treatment with MG-132 proteasomal inhibitor abrogated activin-induced p21 protein decrease, (**Figure 5B**), invoking ubiquitin-mediated proteasomal degradation in activin-induced p21 downregulation. This is akin to UV-induced p21 protein degradation [**24**], but distinct from basal p21 proteasomal degradation [25], which does not employ ubiquitination.

### Nuclear p21 is Lost in a Subset of Primary Colon Cancers with Intact *ACVR2*

**Figure 6:**
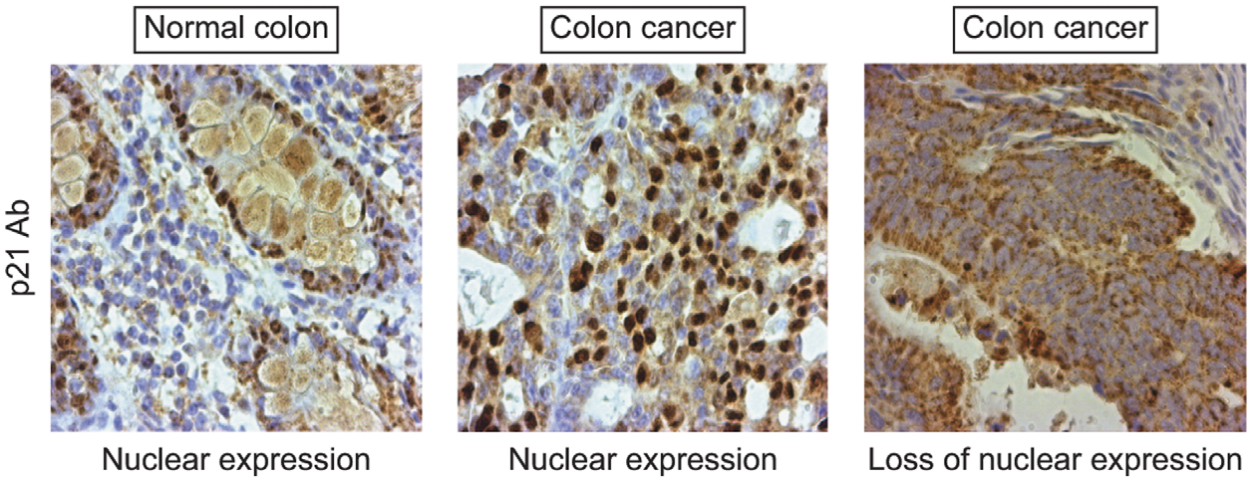
Expression of p21 is lost in a subset of primary colon cancers correlating with the ACVR2/TGFBR2 receptor status. Fifty-six colon cancers were stained for ACVR2, TGFBR2 and p21. Representative examples for p21 staining are shown: normal colon tissue with nuclear staining (left panel), colon cancer sample with maintained nuclear p21 staining (middle panel), and colon cancer sample with loss of nuclear p21 staining (right panel).

We then assessed whether impaired activin/TGFβ signaling affected p21 localization in primary colon cancers. We determined presence versus loss of nuclear p21 expression in 56 primary colon cancer specimens of various genomic subtypes, and correlated this data with the activin and TGFβ receptor status (**Table 1**). We found that a large subset of colon cancers showed loss of nuclear p21, and that this loss was associated with preservation of ACVR2 (**Table 1** and **Figure 6**), suggesting decreased signaling through the SMAD4/p21 axis, but intact activin SMAD4-independent signaling. The opposite was the case for TGFBR2: Preservation of TGFBR2 was associated with persistent nuclear p21 (**Table 1**). This data is consistent with our *in vitro* findings of TGFβ/SMAD4-dependent upregulation of p21 and activin/non-SMAD4-dependent downregulation of p21.

**Table 1:** Nuclear p21 expression in primary colon cancers correlates with ACVR2 and TGFBR2 receptor expression.

A			
p21 expression	ACVR2+ (39)	ACVR2− (17)	p-value
**nuclear** (20)	7	13	0.0001
**loss of nuclear** (36)	32	4	
**B**			
**p21 expression**	**TGFBR2+ (29)**	**TGFBR2− (27)**	**p-value**
**nuclear** (20)	17	3	0.0006
**loss of nuclear** (36)	12	24	

A) ACVR2 expression correlates with loss of nuclear p21 in colorectal cancers:

χ^2^(1, N = 56) = 15.204, p = 0.0001.

B) Loss of TGFBR2 expression correlates with loss of nuclear p21 in colorectal cancers:

χ^2^(1, N = 56) = 11.755, p = 0.0006.

## Discussion

In MSI-H colon cancers, both TGFβ and activin signaling are abrogated due to frameshift mutations in the type II receptor [26]. The loss of both of these signaling pathways may be beneficial and additive for tumor growth [20], [27], but the differential effect on migration remains unclear. TGFβ and activin utilize the same intracellular SMAD proteins (SMAD2/3 and SMAD4) to transmit their signal. Both ligand specific pathways are commonly inactivated in MSI-H colon cancers, for which we previously observed greater than 50% overlap between *ACVR2* and *TGFBR2* mutations [6]. Interestingly, they are less commonly inactivated in MSS colon cancers, which tend to have a worse prognosis than MSI-H colon cancers [9], and both pathways may be targeted independently. Here we show that while activin and TGFβ both can induce growth suppression and apoptosis to varying degrees, they also enhance migration, thus sharing in tumor suppressive as well as cancer promoting properties. Fine-tuning of these opposing effects as well as differential regulation of TGFβ versus activin signaling is likely an important process in carcinogenesis influencing the fate of cancer cells. This manuscript explores the differential effects and regulation of activin and TGFβ signaling in colon cancer.

Here we show that in colon cancer cells, despite identical downstream SMAD signaling, activin and TGFβ have opposing effects on the cdk2 inhibitor p21 resulting in distinct regulations of each pathway. While TGFβ has a strong up-regulatory effect on p21, activin signaling leads to a slight decrease in p21 protein levels. Interestingly, both ligands induce SMAD4-dependent p21-mediated cell growth suppression and cell death, yet TGFβ appears to be a more potent inducer of growth suppression, while activin on the other hand is a more potent inducer of apoptosis. As previously described, both TGFβ and activin enhance cell migration [20], [22]. Notably, we now show that activin’s pro-migratory effect is regulated in a SMAD4-independent fashion and describe for the first time a concomitant increase in p21 ubiquitination and proteasomal degradation. Hence, whereas activin-induced growth suppression is dependent on p21, activin-induced migration is accompanied by reduced p21 levels and independent of SMAD4. While it is known that UV-induced p21 protein degradation is ubiquitinin-dependent [24], basal p21 degradation via the proteasome is not [25]. Recent data implicates ERK2 in mediating nuclear to cytosolic shifting and ensuing ubiquitinin-mediated degradation of p21 [28]. A variety of ubiquitin ligases to include Ecto and Smurf-1 have been found to target both SMAD-dependent and independent TGFβ signaling [29]. The specific ubiquitin ligase responsible for activin-mediated p21 ubiquitination has not been determined to date.

Increase or decrease of p21 levels could drive a cell towards the preferential activation of either the SMAD4-dependent or independent signaling pathway and vice versa, thus modulating the overall cellular response. Conclusively, p21 appears to be an important player for the differential regulation of SMAD4-dependent and independent pathways controlled by activin and TGFβ (**Figure 4**).

In fact, it appears that both activin and TGFβ SMAD and non-SMAD signaling occur simultaneously and that the net effect is a result of the relative context-dependent dominance of a given ligand and/or pathway. Differential regulation of p21 may be an important mechanism to control and fine-tune preferential signaling dependent or independent of SMAD with potential prognostic relevance. Loss of SMAD signaling has been associated with increased migration and loss of growth suppression in colon cancer [22], and we now show one possible mechanism in colon cancer by which SMAD signaling may be bypassed via preferential activin signaling, presenting an explanation for the pro-migratory and pro-proliferative effects accompanying lost SMAD signaling.

p21 plays a complex role in cancer. Tumor suppressive properties of p21 have been described in the context of induction of growth arrest, differentiation and senescence and studies in different cancer types showed that p21 expression correlates with a favorable diagnosis [10]. Consistent with the above finding, several studies in colon cancer revealed an association between p21 downregulation and metastasis as well as poor survival [30], [31], [32] [33], however, some reports point towards a dual role in several cancers with increase of p21 correlating with poor outcome [34], [35].

Here, we report a substantial number of primary colon cancers with loss of nuclear p21, which correlates with presence of ACVR2 and absence of TGFBR2. This is in line with our *in vitro* data where we show downregulation of p21 in the context of enhanced SMAD4-independent signaling induced by activin. It is also consistent with the concept of an absent upregulation of p21 after abrogation of the TGFβ/SMAD4 axis, which can be explained by knockdown of SMAD4, as in our experiments, but also by absence of TGFBR2, as seen in the cancer samples. The functional consequences we would expect from decreased p21 levels in conjunction with preservation of ACVR2 and loss of TGFBR2 based on our data are enhanced migration via SMAD4-independent signaling and loss of growth suppression through the TGFβ/SMAD4/p21 axis. Independent of the effect on growth suppression, which alone has been found to be a weak prognostic marker in many cancers [36], the ACVR2+/TGFBR2- receptor status associated with loss of nuclear p21 points to a pro-metastatic and thus more aggressive cancer phenotype. This is consistent with previous findings showing that loss of p21 is associated with worse outcome in various cancer types [10]. While other signaling pathways may direct p21 localization, our data establish the basis for further assessment of activin and TGFβ receptor status in association with p21 localization for prediction of outcome and response to therapy in colon cancer.

In summary, our data show that TGFβ is a more potent inducer of growth suppression while activin is a more potent inducer of apoptosis. Further, growth suppression and apoptosis by both ligands are dependent on SMAD4 and p21. However, activin downregulates nuclear and total p21 protein in a SMAD4-independent fashion in conjunction with increased ubiquitination and proteasomal degradation associated with enhanced migration. TGFβ on the other hand upregulates nuclear p21 in a SMAD4-dependent fashion to affect growth arrest and may bypass p21 to affect migration. Further, primary colon cancers show differential p21 expression consistent with their *ACVR2/TGFBR2* receptor status. Conclusively, we report p21 as a differentially affected activin/TGFβ target and mediator of ligand-specific functions in colon cancer, which might be exploited for future risk stratification and therapeutic intervention.

## Materials and Methods

### Ethics Statement

This study was conducted according to the principles expressed in the Declaration of Helsinki. The study was approved by the Institutional Review Board of the University of North Carolina hospitals. All patients provided written informed consent for the collection of samples as part of the under IRB approval conducted North Carolina Colorectal Cancer Study (NCCCS) as referenced below. The study was approved by the Northwestern University Institutional Review Board (IRB#STU00020989). Written informed consent was obtained from all participants.

### Patient Samples

Colon tumors were prospectively collected under IRB approval as part of the North Carolina Colorectal Cancer Study (NCCCS), a population-based, case-control study comprising 503 patients [14], [15]. For this study, 15 patient samples with ample tumor and normal tissue were randomly selected. For verification, we collected an additional 41 consecutive colorectal cancer specimens from Northwestern University under institutional IRB approval (IRB#STU00020989) (Table S1). All tumors were formalin-fixed, embedded in paraffin and cut into 5 µm sections.

### Colon Cancer Cell Lines

SW480 cells (ATCC, Manassas, VA) were maintained in Iscove’s Modified Dulbecco’s and FET cells (generous gift from Michael Brattain, University of Nebraska, Omaha, NE [16]) in F12/Dulbecco’s Modified Eagles medium (both Invitrogen, Carlsbad, CA) supplemented with 10% fetal bovine serum and penicillin G [100 U/ml]/streptomycin [100 µg/ml] (Invitrogen). Cells were grown at 37°C in a humidified incubator with 5% CO_2_. All cells were serum starved for 24 hours prior to experimentation to approximate cell cycle synchronization. Cells were tested for mycoplasma infection using the PCR Mycoplasma Detection Set (Takara, Otsu, Japan) and authenticated by STR profiling using the PowerPlex 1.2 System (Promega, Madison, WI).

### Antibodies and Reagents

Activin A was reconstituted in PBS, TGFβ1 in 4 mM HCl according to manufacturer’s instruction (both R&D, Minneapolis, MN) and used at final concentrations of 25 ng/ml and 10 ng/ml as previously described [17], [18], [19], [20]. MG-132 (Calchemie, Darmstadt, Germany) was used for inhibition of the proteasome. For immunohistochemical analyses, we used a goat polyclonal antibody against ACVR2 (1∶50) (ab10595, Abcam, Cambridge, MA), as well as mouse monoclonal antibodies against TGFBR2 (1∶50) (ab78419, Abcam) and p21 (1∶150) (sc-817, Santa Cruz Biotechnology, Santa Cruz, CA). For Western blotting, p21 (# sc-469) (1∶250) (Santa Cruz, Biotechnology), α-tubulin (# 3873), H3 histone (# 9715) (both Cell Signaling Technology, Danvers, MA), and GAPDH (# sc-47724) (all 1∶1000) antibodies (Santa Cruz, Biotechnology) were utilized.

### MTT-Growth Proliferation Assay

Cellular metabolic activity, indicative of the growth status of cells following treatment with activin or TGFβ was assayed using 3-(4,5-dimethylthiazol-2-yl)-2,5-diphenyltetrazolium bromide (MTT) (MP Biomedicals, Aurora, OH) as previously described [20].

### Cell Death/Apoptosis TUNEL Assay/Cellular DNA Fragmentation ELISA

Cells were seeded in 6 well plates at a density of 10,000 cells per well, serum starved for 24 hours and treated with ligand. 24 hours after treatment, cells were lysed with trypsin and counted using a hemacytometer as previously described [21]. Apoptosis was determined using TUNEL staining with APOPTAG In Situ Detection and DAPI counterstaining (Chemicon International/Millipore, Temecula, CA), according to the manufacturer’s instructions.

As an alternative assay of apoptosis, a photometric enzyme-linked immunoabsorbent assay for the detection of BrdU-labeled DNA fragments (Cellular DNA Fragmentation ELISA, Roche, Indianapolis, IN) was used. Both FET and SW480 cells were seeded in 6 well plates at a density of 10,000 cells per well. FET cells were seeded with or without SMAD4 or p21 siRNA. After 24 hours serum starvation, cells were treated with ligand for 24 hours. Apoptosis was determined via BrdU-labeling of intracellular DNA fragments and quantified via anti-DNA antibody detection ELISA according to the manufacturer’s guidelines.

### p21 Luciferase Assay

The pWWP-luc plasmid (generous gift from B. Vogelstein, Johns Hopkins University, Baltimore, MD), containing the promoter of p21^cip1/waf1^, was cotransfected with the *Renilla*-expressing pRL-TK vector (Promega). Luciferase activity was measured 24 hours after transfection by a dual luciferase reporter system (Promega). Relative luciferase activity was normalized to the *Renilla* luciferase activity as previously described [21].

### Quantitative Expression of p21 mRNA

RNA was extracted using the AllPrep DNA/RNA Mini Kit (Qiagen, Valencia, CA). RNA quality was assessed with the Agilent Bio-Chip (RIN >9.5). One microgram of RNA of each sample was reverse-transcribed using the Superscript III First-Strand Synthesis SuperMix and Oligo(dT)20 primers by Invitrogen according to the manufacturer’s instruction. Reverse transcription was followed by RNase H digest (New England Biolabs, Ipswich, MA). Quantitative PCR was carried out using specific primers for p21 (5′-GACTCTCAGGGTCGAAAACG-3′, 5′-GGATTAGGGCTTCCTCTTGG-3′). Each experiment was performed as a standard curve experiment based on five serial dilutions (1∶10), utilizing the Fast SYBR Green PCR MasterMix (Applied Biosystems, Foster City, CA). Each reaction was performed in triplicate using a total reaction volume of 20 µl and a final primer concentration of 100 nM. The experiments were performed and analyzed on the 7900 HT Fast Real-Time PCR System (Applied Biosystems) following the standard protocol and conditions for the Fast SYBR Green Master Mix. A dissociation stage was added to the run protocol to ensure specificity of the detected signal. For normalization purposes expression levels of L19 were determined accordingly in the same run to exclude effects of inter-run variability (5′-ACCCCAATGAGACCAATGAAAT-3′, 5′-CAGCCCATCTTTGATGAGCTT-3′). The relative expression of p21 normalized to L19 levels was calculated for each sample and plotted on a graph.

### Total Lysis, Nuclear/Cytosolic Separation, and Western Blotting

Cells were lysed using total lysis buffer RIPA (1% NP40, 0.1% SDS, 1% DCA, 50 mM Tris HCl pH 7.2) with added protease and phosphatase inhibitors as previously described [20], [21]. Cytoplasmic and nuclear fractions were extracted with NE-PER Nuclear and Cytoplasmic Extraction Reagents (Thermo Fisher Scientific, Rockford, IL) according to the manufacturer’s instruction. Western blotting was performed using standard protocols with 4–20% polyacrylamide gels, nitrocellulose membrane transfers, overnight incubation with primary antibody at 4°C followed by horseradish peroxidase-linked secondary antibodies (Santa Cruz Biotechnology, Santa Cruz, CA) and detection by ECL (Amersham, Little Chalfont, UK) [20], [21], visualization, and quantification of chemiluminescence with the LAS-3000 (FujifilmUSA, Valhalla, NY).

### siRNA and Transfection

Two specific siRNAs for each p21 and SMAD4 (Ambion, Austin, TX and Santa Cruz Biotechnology) were transiently delivered at a final concentration of 10 nM via electroporation using the AMAXA Nucleofector (Lonza, Basel, Switzerland) in 12-well plates at a density of 2×10^6^ according to the manufacturer’s instructions. Transfection efficiency was confirmed using the pmaxGFP™ Control Vector (Lonza). Forty-eight hours post transfection, colon cancer cells were lysed for subsequent RNA and protein extraction.

### Migration/Invasion Assay

Migration assays were performed as previously described [20]. Briefly, Corning Costar Transwell 12 well plates (8 µm pores, Corning, NY) with fibronectin or matrigel (Sigma, St. Louis, MO) were seeded with colon cancer cells with or without ligand in the presence or absence of siRNA. Cells were then allowed to migrate for 4 hours, stained, and images were captured using an Axiovert 2000 microscope with an AxioCAM HRC Camera (both Zeiss Microimaging, Thornwood, NY). Images from 5 microscopic fields at the center of each well were counted.

### Immunohistochemistry for ACVR2, TGFBR2, p21 Expression and Localization

Slides containing primary colon cancer tissues were processed as previously described [6] and stained for ACVR2, TGFBR2, and p21 using the Catalyzed Signal Amplification System (CSA) by DAKO (Carpinteria, CA). ACVR2 and TGFBR2 staining was grouped into negative (no or weak signal) and positive (moderate or strong signal) receptor status. The percentage of p21 positive nuclei in each cancer samples was assessed. Tumors with more than 50% of p21 positive nuclei were scored as nuclear positive cancers. Slides were scored in a blinded fashion by two investigators. Both investigators had to be in agreement for a tumor to be called negative.

### Immunoprecipitation, Ubiquitination Assay, and Proteasomal Inhibition

Proteasomal inhibition was achieved by pre-treatment of cells with 10 µM of MG-132 (Calchemie) for 30 minutes. 80 µg of total protein lysate from various treatments were incubated with 1 mg of p21 antibody (# sc-469, Santa Cruz Biotechnology) overnight at 4°C. Protein G beads (Invitrogen, Carlsbad, CA) were added for 4 hours. After denaturation with sample buffer, Western blotting was performed on 4–20% gradient gels (Biorad, Hercules, CA) and nitrocellulose membranes after boiling for 15 minutes incubated with antibodies against ubiquitin (1∶1000) (# sc-8017) antibody (Santa Cruz Biotechnology), p21 and IgG (# 111-035-008; Jackson ImmunoResearch, West Grove, PA) or GAPDH (# sc-47724; Santa Cruz Biotechnology) as respective controls.

### Statistical Analysis

Differences between two respective groups were determined using Student’s *t*-test. Probability values less than 0.05 were considered to be significant. Data shown represents repeated experiments on multiple biological replicates. For association of p21 localization with receptor status, we performed Chi-Square test calculations using the MedCalc® software (Version 11.6.1).

## Supporting Information

Figure S1 **p21 mediates TGFβ-induced growth suppresion and counteracts TGFβ-induced SMAD4-independent migration in the presence of SMAD4.** A) FET cells were treated with either scramble (SC) or p21 specific siRNA. Growth suppresion was assessed by MTT-metabolic assay following TGFβ treatment. TGFβ induced cell grwoth inhibition in the presence of p21, but the effect was reversed in the absence of p21. B) Total viability is decreased in *SMAD4*-wild type colon cancer cells following TGFβ treatment in the presence of p21. FET cells were treated with either scramble (SC) or p21 specific siRNA. Cell viability was assessed by trypan blue staining following TGFβ treatment. Trypan blue positiv cells after TGFβ treatment were decreased in presence of p21, but increased after p21 knockdown. C) TGFβ induced cell migration in SMAD4-positiv and SMAD4-negativ cell lines. Cellular migration is induced in *SMAD*4-wild type FET cells and *SMAD4*-null SW480 cells following TGFβ treatment, but more pronounced induction of migration is seen in the absence of SMAD4. D) p21 knockdown increased TGFβ-induced migration in FET cells. Loss of in the absence of SMAD4 does not further increase migratory induction (*p<0.05, **p<0.01, ***p<0.001).
(TIF)

Table S1 **Characteristics of colon cancer patient cohort randomly selected from North Carolina Colorectal Cancer Study (NCCCS) (19, 20) (patients 1–15) and NW cohort (patients 16–56) for p21 staining.** Four patients from the NW cohort did not have a stage information available (X).
(DOC)
