# Supplementary figures and images for: Effects of Activin and TGFβ on p21 in Colon Cancer
Source: PLoS One. 2012 Jun 26;7(6):e39381. doi: 10.1371/journal.pone.0039381 (PMC3383701; doi:10.1371/journal.pone.0039381)

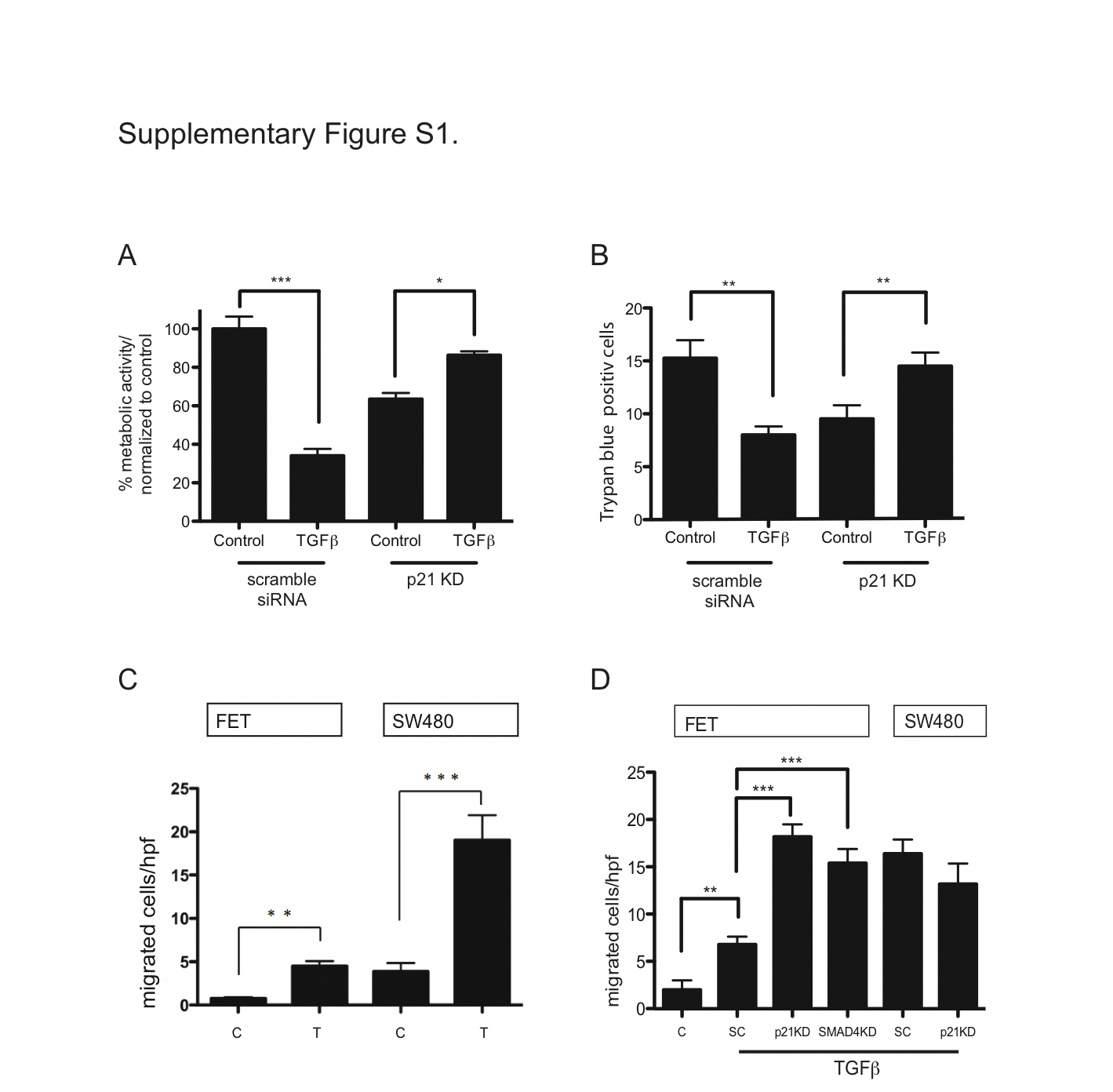

Supplement: Figure S1 — p21 mediates TGFβ-induced growth suppresion and counteracts TGFβ-induced SMAD4-independent migration in the presence of SMAD4. A) FET cells were treated with either scramble (SC) or p21 specific siRNA. Growth suppresion was assessed by MTT-metabolic assay following TGFβ treatment. TGFβ induced cell grwoth inhibition in the presence of p21, but the effect was reversed in the absence of p21. B) Total viability is decreased in SMAD4-wild type colon cancer cells following TGFβ treatment in the presence of p21. FET cells were treated with either scramble (SC) or p21 specific siRNA. Cell viability was assessed by trypan blue staining following TGFβ treatment. Trypan blue positiv cells after TGFβ treatment were decreased in presence of p21, but increased after p21 knockdown. C) TGFβ induced cell migration in SMAD4-positiv and SMAD4-negativ cell lines. Cellular migration is induced in SMAD4-wild type FET cells and SMAD4-null SW480 cells following TGFβ treatment, but more pronounced induction of migration is seen in the absence of SMAD4. D) p21 knockdown increased TGFβ-induced migration in FET cells. Loss of in the absence of SMAD4 does not further increase migratory induction (*p<0.05, **p<0.01, ***p<0.001). (TIF) [file pone.0039381.s001.tif]
