# Supplementary material for: Effects of Activin and TGFβ on p21 in Colon Cancer
Source: PLoS One. 2012 Jun 26;7(6):e39381. doi: 10.1371/journal.pone.0039381 (PMC3383701; doi:10.1371/journal.pone.0039381)
Supplement: Table S1 — Characteristics of colon cancer patient cohort randomly selected from North Carolina Colorectal Cancer Study (NCCCS) (19, 20) (patients 1–15) and NW cohort (patients 16–56) for p21 staining. Four patients from the NW cohort did not have a stage information available (X). (DOC) [file pone.0039381.s002.doc]

**Supplementary Table S1.**

| Patient# | Gender | Age | Stage | TGFBR2 | ACVR2 |
| --- | --- | --- | --- | --- | --- |
| 1 | F | 63 | II | + | + |
| 2 | F | 82 | III | + | + |
| 3 | M | 57 | II | + | - |
| 4 | M | 71 | I | + | - |
| 5 | M | 68 | III | + | - |
| 6 | F | 75 | II | - | + |
| 7 | M | 80 | IV | - | + |
| 8 | M | 65 | II | - | + |
| 9 | F | 66 | III | - | + |
| 10 | F | 70 | II | - | + |
| 11 | M | 88 | IV | - | + |
| 12 | M | 62 | III | - | + |
| 13 | F | 78 | II | - | + |
| 14 | M | 91 | IV | - | + |
| 15 | M | 85 | III | - | + |
| 16 | F | 70 | II | + | + |
| 17 | F | 71 | III | + | + |
| 18 | F | 71 | IV | + | + |
| 19 | F | 40 | III | + | + |
| 20 | F | 55 | III | + | + |
| 21 | F | 55 | II | + | + |
| 22 | M | 78 | II | + | + |
| 23 | F | 34 | IV | + | + |
| 24 | M | 79 | III | + | + |
| 25 | M | 80 | I | + | + |
| 26 | F | 50 | IV | + | + |
| 27 | M | 73 | III | + | + |
| 28 | M | 82 | X | + | + |
| 29 | M | 49 | X | + | + |
| 30 | M | 49 | IV | + | + |
| 31 | F | 62 | III | + | - |
| 32 | M | 47 | III | + | - |
| 33 | M | 80 | III | + | - |
| 34 | F | 74 | III | + | - |
| 35 | M | 48 | III | + | - |
| 36 | M | 63 | X | + | - |
| 37 | F | 70 | I | + | - |
| 38 | F | 72 | IV | + | - |
| 39 | F | 52 | IV | + | - |
| 40 | F | 70 | III | - | + |
| 41 | F | 57 | II | - | + |
| 42 | M | 37 | II | - | + |
| 43 | M | 41 | X | - | + |
| 44 | M | 65 | III | - | + |
| 45 | M | 65 | IV | - | + |
| 46 | M | 45 | IV | - | + |
| 47 | F | 85 | I | - | + |
| 48 | F | 72 | I | - | + |
| 49 | M | 37 | I | - | + |
| 50 | F | 58 | IV | - | + |
| 51 | M | 82 | IV | - | + |
| 52 | M | 78 | III | - | - |
| 53 | M | 63 | III | - | - |
| 54 | M | 69 | III | - | - |
| 55 | M | 64 | III | - | - |
| 56 | F | 84 | IV | - | - |
